# Chemical Science Research, Elementary School Children and Their Teachers Are More Closely Related than You May Imagine: The “I Bet You Did Not Know” Project

**DOI:** 10.1021/acs.jchemed.3c00233

**Published:** 2024-01-19

**Authors:** Alison J. Trew, Craig Early, Rebecca Ellis, Julia Nash, Katharine Pemberton, Paul Tyler, Timothy G. Harrison, Dudley E. Shallcross

**Affiliations:** † Primary Science Teaching Trust, 12 Whiteladies Road, Bristol, BS8 1PD, U.K.; ‡ School of Chemistry, Cantock’s Close, University of Bristol, Bristol, BS8 1TS, U.K.; § Department of Chemistry, University of the Western Cape, Robert Sobukwe Road, Bellville, 7535, South Africa

**Keywords:** Elementary, Middle School Science, Public Understanding of Science, Outreach, Analogies, Transfer, Hands-On Learning, Inquiry-Based, Discovery Learning, Learning Theories

## Abstract

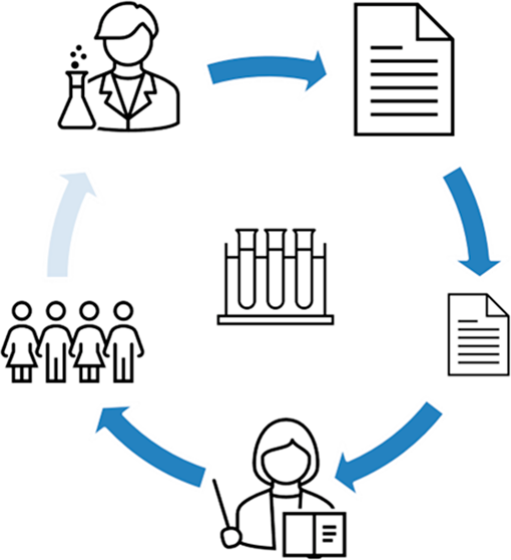
Topics associated with the chemical sciences form a significant part of the curriculum in science at the primary school level in the U.K. In this methodology paper, we demonstrate how a wide range of research articles associated with the chemical sciences can be disseminated to an elementary school audience and how children can carry out investigations associated with cutting-edge research in the classroom. We discuss how the Primary Science Teaching Trust’s (PSTT’s) “I bet you did not know” (IBYDK) articles and their accompanying Teacher Guides benefit children, primary (elementary) school teachers, and other stakeholders including the researchers themselves. We define three types of research articles; ones describing how children can reproduce the research themselves without much adaptation, others where children can mirror the research using similar methods, and some where an analogy can be used to explain the research. We provide exemplars of each type and some preliminary feedback on articles written.

## Introduction

In addition to the accepted roles of school and the tasks befalling teachers (e.g., teaching fundamental numeracy, literacy and other skills, elements of critical thinking, social and citizenship skills), a major challenge for elementary and secondary teachers is to prepare children, currently in school, for jobs in the future^1^ including many that do not currently exist.^2^ The challenge is perceived to be even harder for children and their teachers in science in the elementary (primary in the U.K.) school environment given the supposed lack of background science knowledge for both teachers and children. In the case of chemistry related materials, the concern about health and safety when handling chemicals is an additional barrier for all school groups but, in particular, the elementary school one. Making current research articles available to the elementary school arena, in forms that support elementary school science principles and concepts goes someway to addressing the challenge posed.^3,4^ Elementary school children and their teachers could engage with current research, gaining science capital^5^ through being introduced to science careers and scientists, and by carrying out similar investigations they may begin to see themselves as scientists.^6^

Outreach to elementary school students, especially in Chemistry, is limited. The use of “chemicals” in elementary school raises several potential issues with health and safety and is a reason why the number of chemistry-based outreach programs to primary schools is small.^7−13^ The elements of the U.K. curriculum that are associated with Chemistry include properties of materials, rocks, states of matter, and the Earth, where we would include the gases present in the atmosphere. Some of the topics in this already short list overlap between Chemistry and Physics of course. Typically, investigations involving red cabbage indicator,^9^ hard and soft solids^11^ and those involving taste and smell of food stuffs are ones that students may undertake. Others that involve inspection of objects, such as rock types, are more passive in nature. The Bristol ChemLabS Centre for Teaching and Learning^14,15^ have run a circus of experiments involving the iodine clock reaction, reaction of magnesium ribbon with dilute acid and polymer generation.^9^ These experiments require the use of laboratory coats and safety spectacles and the transformation of an elementary school hall into a laboratory (floor protection, etc.).

In this article, we describe some recent chemistry related research articles that have been used to develop curricular materials for the primary classroom in the project, “I bet you did not know” initiated by the UK charity, the Primary Science Teaching Trust (PSTT).^16^ We discuss the way that supporting materials are developed and some feedback on these from teachers and their pupils.

## The “I Bet You Didn”T Know” Methodology

In previous papers^3,4,6,17^ we have described a methodology for sharing cutting-edge research from across all science subjects and creating resources for elementary teachers that are freely downloadable on the PSTT Web site (https://pstt.org.uk/resources/curriculum-materials/cutting-edge-science-primary-schools). This project involves elementary school teachers who have won the UK Primary Science Teacher of the Year Award (7) and as a result have become Fellows of the PSTT virtual College (www.pstt.org.uk). These outstanding teachers of science at elementary level, who are part of the “*I bet you did not know*” project, are also former research scientists, each having undertaken doctoral research with some carrying out postdoctoral research in a range of biological and chemistry related subjects. Therefore, these teachers are ideally placed to connect research articles with elementary school curricula and understand the ideas and concepts that make sense to this school group. The group have access to the research literature through a fellowship at a UK Higher Education Institute and can collect suitable peer-reviewed papers published within the previous two years for consideration. The team meets biannually to review the papers gathered for suitability, the most important factor being a link to concepts taught at elementary school. Papers chosen are then assigned to team members and they will develop an IBYDK article. These are two to three pages that explain the cutting-edge research in language that elementary children can understand and include a glossary of scientific terms that may be unfamiliar. The articles explain what the scientists have done, suggest questions for children to consider and describe related activities that children can do.

**Figure 1:**
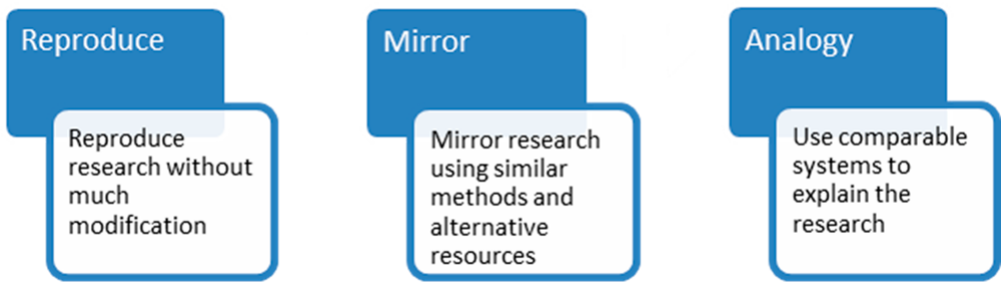
Practical activities and investigations suitable to the project are related to scientists’ cutting-edge research. They fall into three categories: Reproduce the research, Mirror the research, or Analogy, where an analogy is used to explain the research.

So far, three approaches have been used in the development of an article (Figure 1). First, the research can be reproduced without much modification. Although it is unlikely in most cases that a study based in the chemical sciences can be translated straight into an elementary school arena, some that are citizen science type investigations involving counting, collating, or pattern seeking may be possible (4). Second, it may be possible to “mirror” aspects of the research using similar methods in a primary school setting. The scientists’ resources may not be available, but children can carry out investigations using alternative resources and following a similar methodology. Third, for many research papers, it is impossible to reproduce the research as presented, but analogies exist in primary school teaching. Using analogous systems appropriate to the elementary science curriculum, the research can be explained through models, practical activities, or investigations. We illustrate examples of each of these types in the following sections that follow.

**Figure 2:**
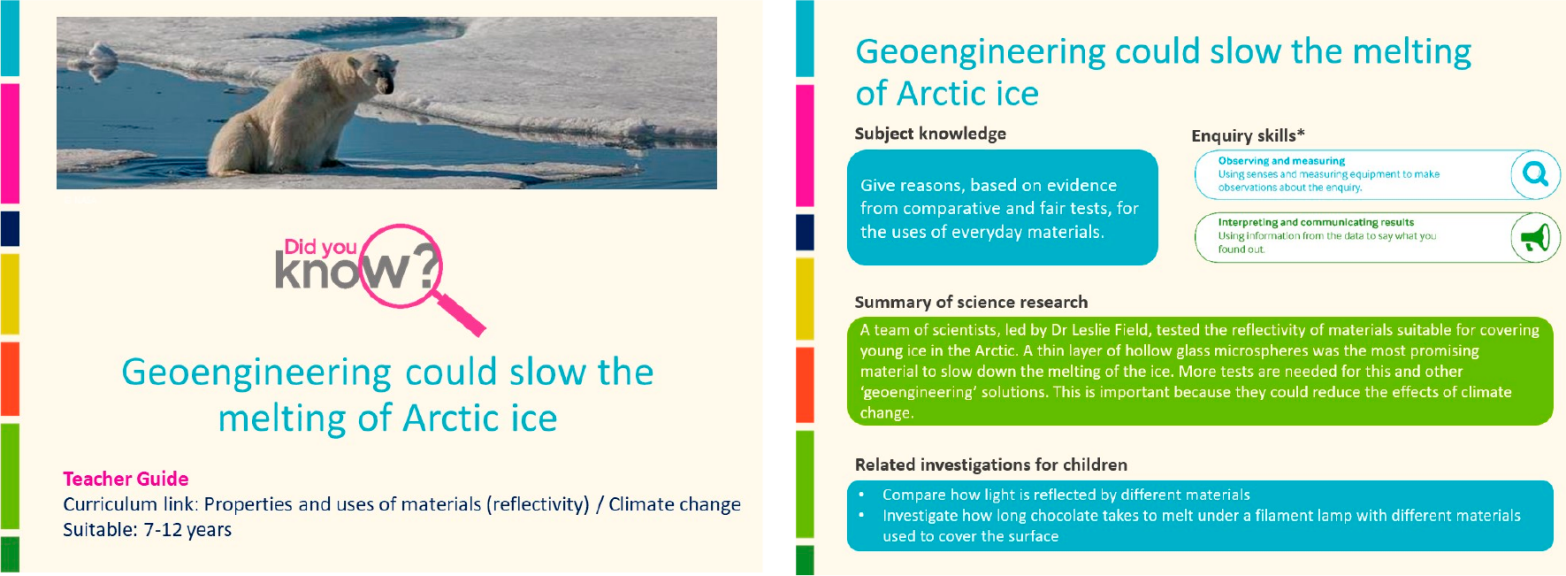
Example of new “*Did you know?*” Teacher Guide showing the primary science curriculum link on the cover slide and learning outcomes for both subject knowledge and skills on the following slide.

In addition to the *I bet you did not know* article, the project provides Teacher Guides (slideshows with teacher notes) that help the teacher to prepare for and deliver the lesson or lessons (see Web site). The Teacher Guides are a key element of the success of the project to date and help teachers (irrespective of their confidence in teaching science) include cutting-edge research as part of a lesson or series of lessons with young children. On the first slide of every Teacher Guide, the link to the curriculum science topic is stated. Then, below each slide are notes for teachers, which include possible learning outcomes, key science vocabulary, and questions to ask children to promote learning. Over the years, more information has been put into the Teacher Guides and we are aware that for some nonspecialist science teachers, this could be too much. An example, **Geoengineering could slow the melting of Arctic ice**https://pstt.org.uk/download/7081/?tmstv=1698234193 is shown in Figure 2. In the Supporting Information, we list all articles produced as of November 2023.

## Chemistry-Based Exemplars

### Research without Much Modification (“Reproduced”): Which Facemask You Should Wear (UK Curriculum Areas; Materials and Their Uses and Separating Materials).

This *I bet you did not know* article^18^ (https://pstt.org.uk/download/2749/?tmstv=1676994610) was inspired by two papers by Zangmeister and co-workers^19,20^ who researched the “Filtration Efficiencies of Nanoscale Aerosol by Cloth Mask Materials Used to Slow the Spread of SARS-CoV-2” and “Hydration of Hydrophilic Cloth Face Masks Enhances the Filtration of Nanoparticles”. The researchers assessed the spread of liquid droplets using nanometer-sized aerosols and compared the effectiveness of different materials against this. In a similar way, children in elementary schools can also create aerosols and use them to investigate the effectiveness of different materials as filters and their suitability for use in making face masks.

The researchers tested some medical filters such as surgical masks, a HEPA vacuum bag, a coffee filter, and a paper towel. In the teacher resource, a suggested question that can be posed to the children is, “Which of these filters do you think will be most effective in preventing the spread of the coronavirus and why?” The children could carry out a range of similar investigations prompted by further questions such as

- What do you think is the best fabric for a facemask? Why?
- What do you think is the best shape for a face mask? Why?
- How many layers do you think are needed? Is there a maximum?
- Can you carry out an investigation to find out?

**Figure 3:**
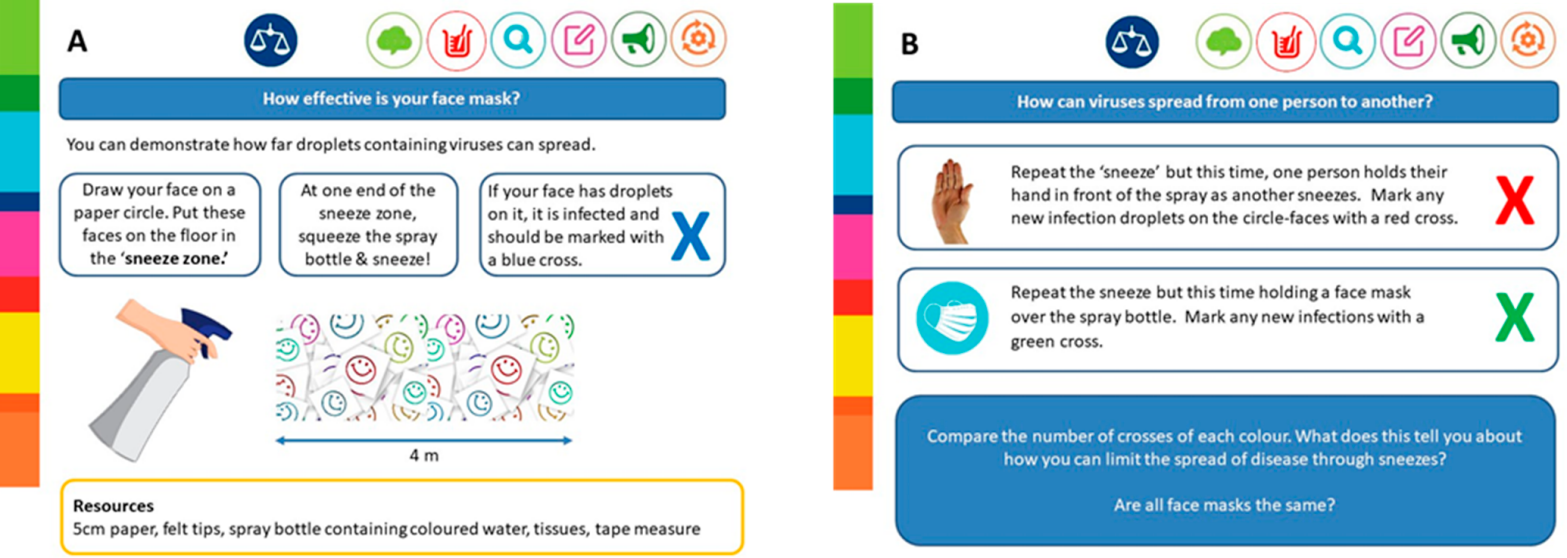
An investigation described in the “Which face mask you should wear” Teacher Guide allowing elementary children to reproduce the scientists’ research: A, finding out how far an aerosol travels; B, testing the effectiveness of fabrics and facemasks on limiting the distance an aerosol travels.

**Figure 4:**
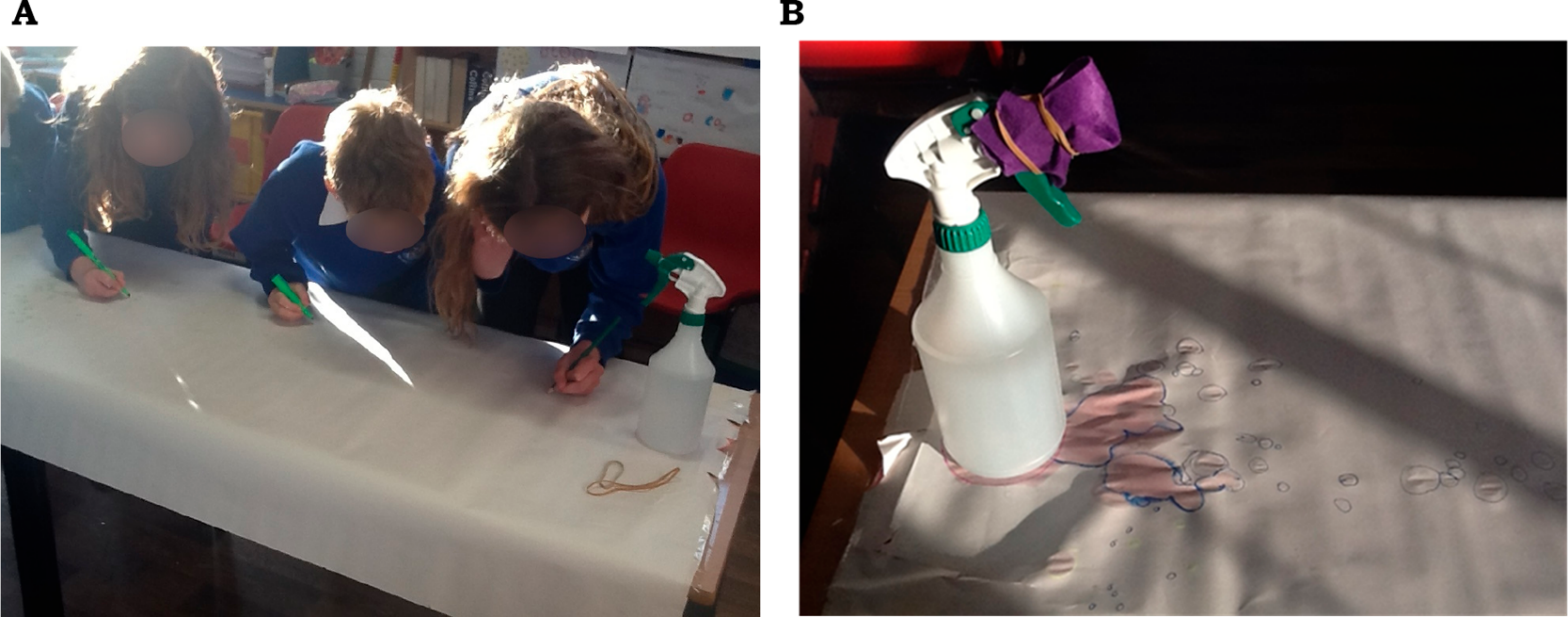
A: Children recording and measuring how far the aerosol reaches; B: The aerosol is covered with fabric ready for testing again.

Supported by the Teacher Guide (Figure 3), teachers have challenged children (ages 9–11) to investigate how effective different fabrics and different facemasks are at preventing the spread of colored water droplets (Figure 4a) and with a covering of the test fabric (Figure 4b). Such an investigation allows children to develop enquiry skills (planning, recording, interpreting and communicating results and evaluating the success of the approach), deepen their knowledge and understanding of a particular topic (in this case separating substances by filtering), as well as feel like they are as scientist themselves carrying out current research.

### Research Where Aspects Can Be Reproduced (“Mirrored”): How to Clean Water Using a Sieve! (UK Curriculum Areas; Materials, Separating Mixtures, and Dissolving)

**Figure 5:**
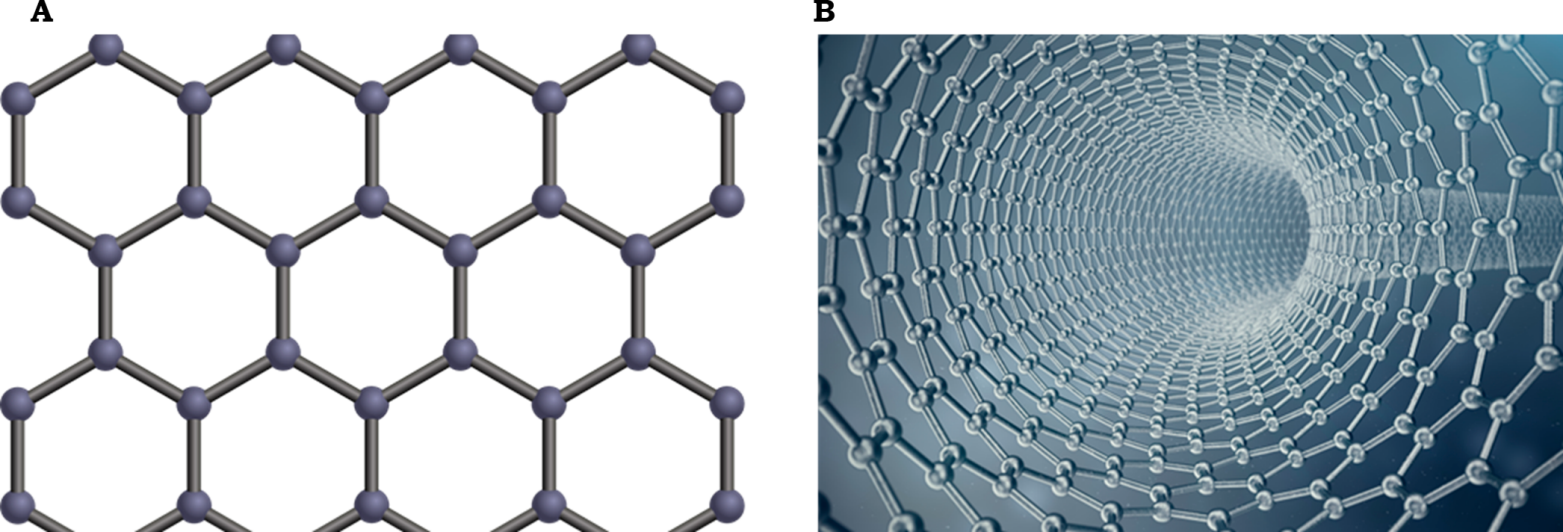
A, Graphene; B, Graphene oxide layer folded into tubes to create a molecular sieve.

This *I bet you did not know* article^21^ (https://pstt.org.uk/download/2877/?tmstv=1679326686) was inspired by “Tunable sieving of graphene oxide membranes”.^22^ The researchers have modified graphene oxide membranes with a view to using these in desalination. Children can understand how to generate graphene sheets (Figure 5a) based on their shape and how these can be folded to form tubes (Figure 5b). They can imagine that the size of the tube can allow certain particles through that are small enough and stop others from becoming too large. They can experiment themselves with macro-sieves that are readily available (e.g., cheese graters or sieves used in cooking with different sieve sizes) and think about items that these sieves can separate from a mixture (e.g., different sized beads). By carrying out their own investigations using macro-sieves, the children can understand that these ideas can be used on a molecular level.

**Figure 6:**
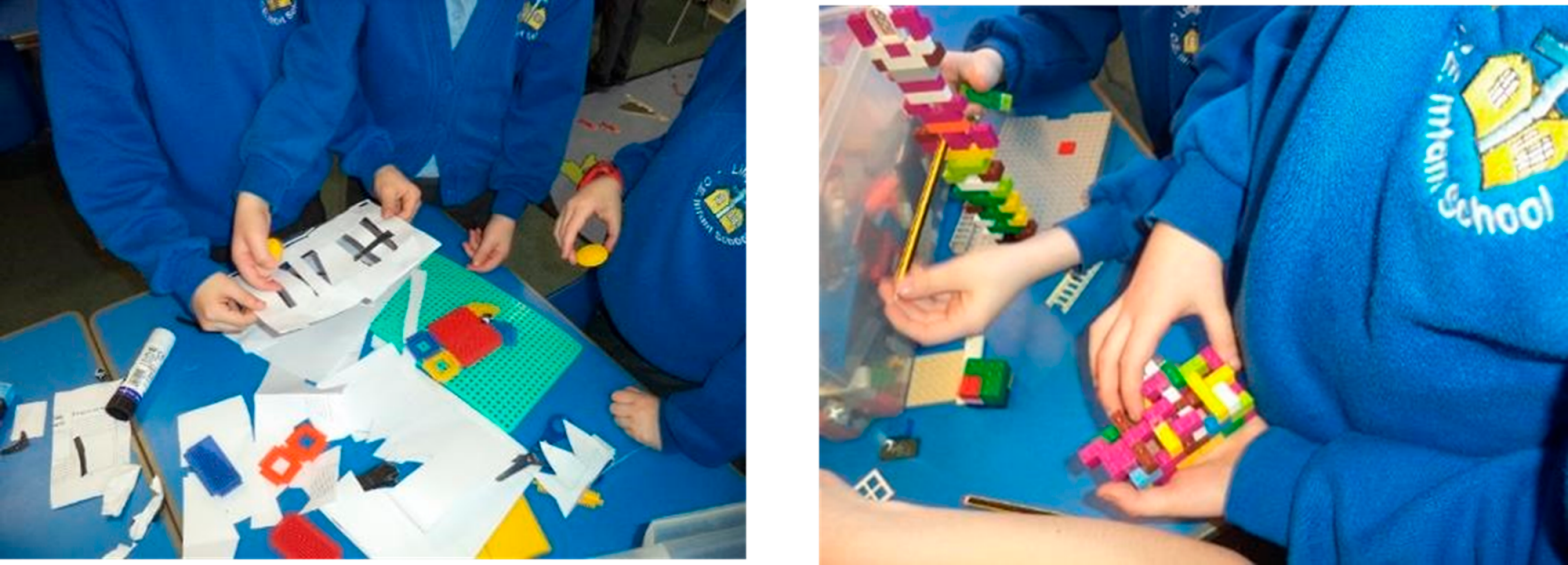
Children make sieves from paper and card and construction toys to separate different size objects.

**Figure 7:**
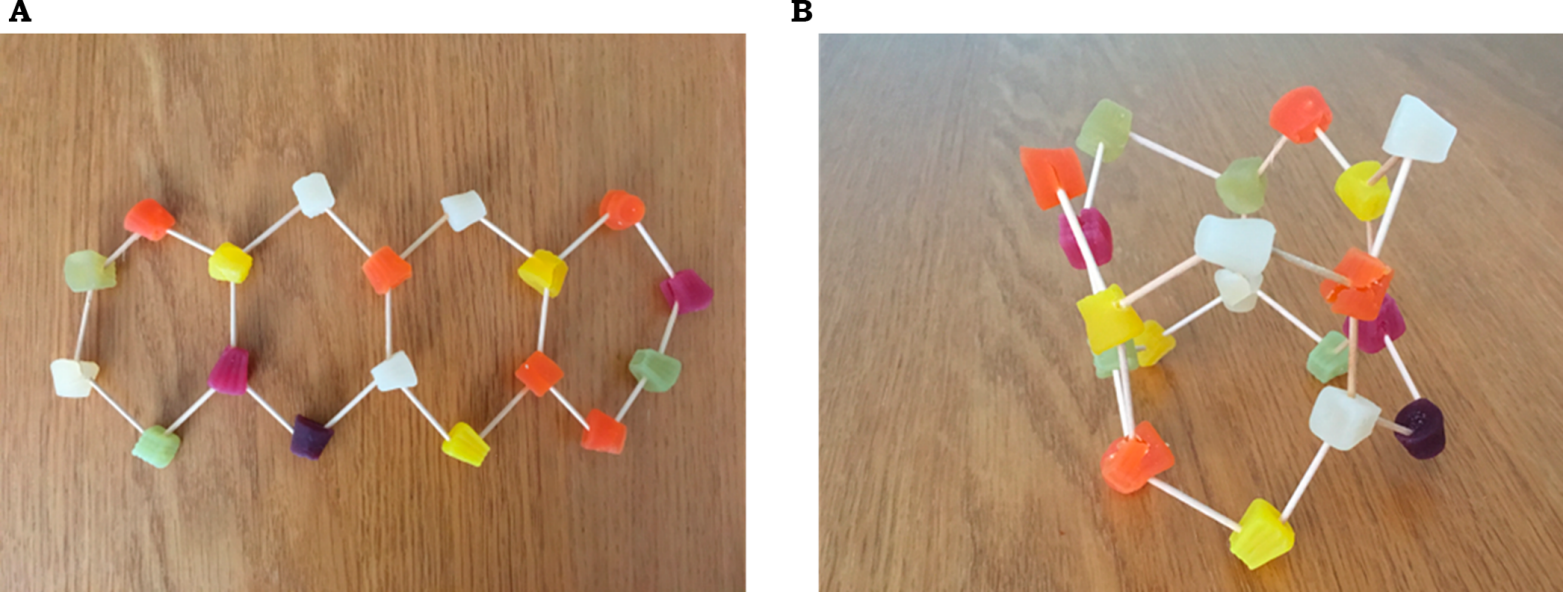
A: Molecular model of graphene; B: A molecular model of a graphene oxide tubular sieve.

In the classroom, it is possible for children to create models of this concept to use to demonstrate their understanding. For example, children (aged 6) have used paper, card and construction toys to create sieves that can separate a mixture of differently sized objects (Figure 6). Older children could make “molecular” models of graphene sheets (Figure 7a) and graphene-based tubes (Figure 7b).

### Using Analogous Systems to Explain the Research (“Analogy”); It Is Raining All over the World - Extreme Weather Connections (UK Curriculum Areas; Weather, States of Matter, and Climate Science)

**Figure 8:**
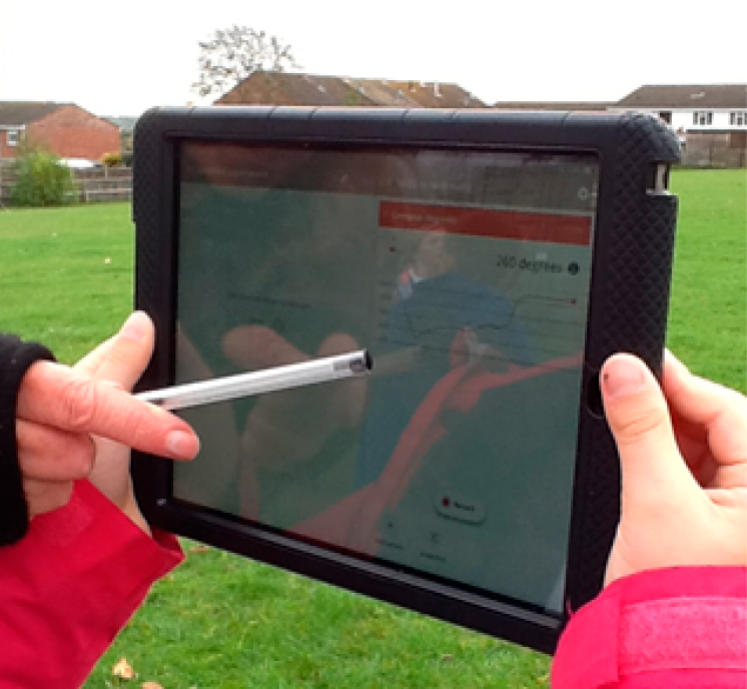
Children (age 10–11) measured the outside temperature, identified clouds, observed trees using the Beaufort scale to decide on wind speed, used an app to measure air pressure, and used bubbles to work out wind direction.

This *I bet you did not know* article^23^ (https://pstt.org.uk/download/2717/?tmstv=1676993941) was based on “Complex networks reveal global patterns of extreme-rainfall teleconnections” by Niklas Boers’ team.^24^ Using satellite data, scientists have shown that global weather events are more closely linked than previously thought. For example, the monsoon systems of south-central Asia, east Asia, and Africa are significantly synchronized. Children do not have the ability to analyze such data, but they can appreciate the type of data and how it is collected through carrying out their own survey of weather (Figure 8). We provide a detailed description of the type of analysis that can be carried out by children in the Supporting Information document, first to reproduce the extreme rainfall in a class-based experiment and then to discuss how to carry out other weather-related activity. An analogous experiment that can be carried out involves an ice cube tray and a beaker of water. Pouring the same volume of water into the tray slowly and very quickly produces very different distributions of water. The slow pour collects water close to where the water is poured with some overflow into neighboring ice cube holders. However, pouring rapidly causes water to be dispersed over a much wider distribution. In the weather study this is what is happening, more intense rain (similar overall amount) over a short period causes connections with areas much further away (see the video on slide 18 of the teacher guide found at https://pstt.org.uk/resources/i-bet-you-didnt-know/?_sft_science_topics=climate-science).

## Impact on Teaching and Learning

Feedback from the children reading IBYDK articles and carrying out related investigations was very positive. Children (all aged 10) who read “Which facemask you should wear” said:

*It was interesting learning about what scientists are doing.*

*I liked learning about what scientists are doing now. It helps us know what scientists actually do, because it will make us ready to be a scientist when we are older.*

*I enjoyed learning about masks in science today—it helped me understand why masks help to keep us safe.*

Younger children who read “How to clean water using a molecular sieve” also shared positive comments:

*I enjoyed doing the sieve because we worked out if other materials could make a brilliant sieve.*

Child (age 6)

Children who read “It is raining all over the world–extreme weather events” were pleased to learn more about climate change and its potential effects. A teacher who shared this article with a class of 10 year olds during COP26 in November 2021 reported:

*Twenty-three children in the class reported that they had explained to their parents/tutor/friend what COP26 was all about last night!*

Other teachers have also provided positive feedback about the resources that we have created:

*I love these and like to use them when I can...please keep releasing them as they are great for both confident and less confident science teachers as you cannot go wrong! You can also use as little or as much of each article as you wish.*

Primary teacher in Wales

*The children...liked the fact that they could see the scientists that were involved in the research, and they knew their name; this made it more real to the children and helped to relate to the learning. The children engaged in meaningful conversations with each other, and they made links to other areas of the curriculum that we had previously looked at. They asked questions, and I know that they continued talking about it at home with their parents after a number of them mentioned at a parents evening consultation. The additional information on the notes of the slides was really helpful, and I used it as a reference when I was teaching the lesson. The use of real data is also very helpful and again makes the learning more meaningful.*

Primary teacher in Kent, England

The *I bet you did not know* articles have been used with children from 5 to 11+ years old. Generally, the articles are presented to the children, and associated activities are undertaken. For the eldest children present at primary school (ages 10–11 years), teachers have told us that some children read the articles independently. Therefore, in response to this feedback, we changed the style of subsequent articles to include a glossary of science vocabulary. In this way, the articles become more accessible to older children as well as their teachers. In the future, we intend to write articles with a lower reading-age requirement, so they are suitable for more children to read alone.

In some examples, children and their teachers have been able to set up an online call with scientists involved in the research. In these sessions they have asked questions (some very insightful), described the research they (the children) have carried out, discussed this with the scientists, and also had a virtual tour of research laboratories where appropriate. In cases where we have observed teachers using these resources, it has prompted much excitement and enthusiasm, and there are examples of children who do not normally engage finding their voice and demonstrating deep understanding of the science supporting the topic investigated. The number of downloads (see Supporting Information) is an indication of the uptake of these articles. Articles that have been available for more than 3 years have typically been downloaded over 1000 times, and while we have no record of who has downloaded them, from formal and informal feedback, we know that a growing number of teachers are incorporating these into lessons. As stated already, connecting with actual scientists and research that they have recently carried out is well received by children, particularly when there is a diverse group of researchers.

## Limitations to This Study

Where we have had extensive feedback and supported teachers in class to use these resources, we see a very positive impact on both the teacher and class. Where a context captures the children’s imagination, helping them to visualize the research undertaken, there is a buzz in the class. However, we are aware that this type of resource is likely to be used by teachers who are confident in teaching science and who are looking for ways to investigate and understand items in the curriculum. That said, we have had feedback from teachers running science clusters where less confident science teachers have been introduced to these resources and found them to be useful. Therefore, we suspect that support for less confident teachers is required to help them use these resources, but the teacher guides that support these resources have received good feedback from all who have engaged.

## Summary

We have shown that it is possible to use cutting-edge research based on chemical sciences in an elementary school setting. Feedback from teachers suggests that the *I bet you did not know* articles are accessible for nonspecialist elementary science teachers and can be shared with a young audience. Feedback from children indicates that learning about cutting-edge research is interesting to children and that the related science activities (whether children reproduce the research, mirror the research, or use analogies to understand cutting-edge research) provide a memorable experience which is likely to have a positive impact on children’s science capital.

## Supporting Information Available

List of all articles, their curriculum links, url and number of times they have been downloaded by November 2023. An extended description of the teacher guide that accompanies the exemplar article “It is raining all over the world” (PDF), (DOCX)
