# Supplementary material for: Chemical Science Research, Elementary School Children and Their Teachers Are More Closely Related than You May Imagine: The “I Bet You Did Not Know” Project
Source: J Chem Educ. 2024 Jan 19;101(2):337–43. doi: 10.1021/acs.jchemed.3c00233 (PMC10867834; doi:10.1021/acs.jchemed.3c00233)
Supplement: Supplementary file 1 — ed3c00233_si_001.pdf [file ed3c00233_si_001.pdf]

SUPPORTING INFORMATION FOR THE PAPER

Chemical Science Research, Elementary School Children and their Teachers are more closely related than you may imagine; The 'I bet you didn't know' project.

Alison J. Trew[1*], Craig Early[1], Rebecca Ellis[1], Julia Nash[1], Katharine Pemberton[1], Paul Tyler[1], Timothy G. Harrison[2#] & Dudley E. Shallcross[2,3*]

1. Primary Science Teaching Trust. 12 Whiteladies Road, Bristol, BS8 1PD, U.K.

2. School of Chemistry, Cantock's Close, University of Bristol, Bristol, BS8 1TS, UK.

3. Department of Chemistry, University of the Western Cape, Robert Sobukwe Road, Bellville, 7535, South Africa,

Corresponding authors Alison J. Trew (alison.trew@pstt.org.uk) and Dudley E Shallcross (d.e.shallcross@bris.ac.uk)

OVERVIEW

We present an extended description of the teacher guide that accompanies the exemplar article 'It is raining all over the world' and a table summarising all the articles written as of November 2023 and weblinks correct at the time of submission.

Extreme weather teacher guide example[S1,S2]

Figure S1 provides teaching input on new subject knowledge.

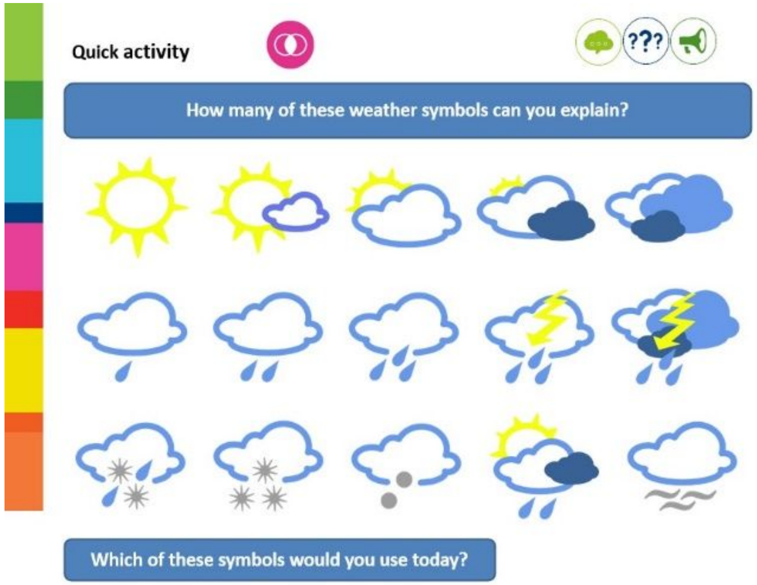

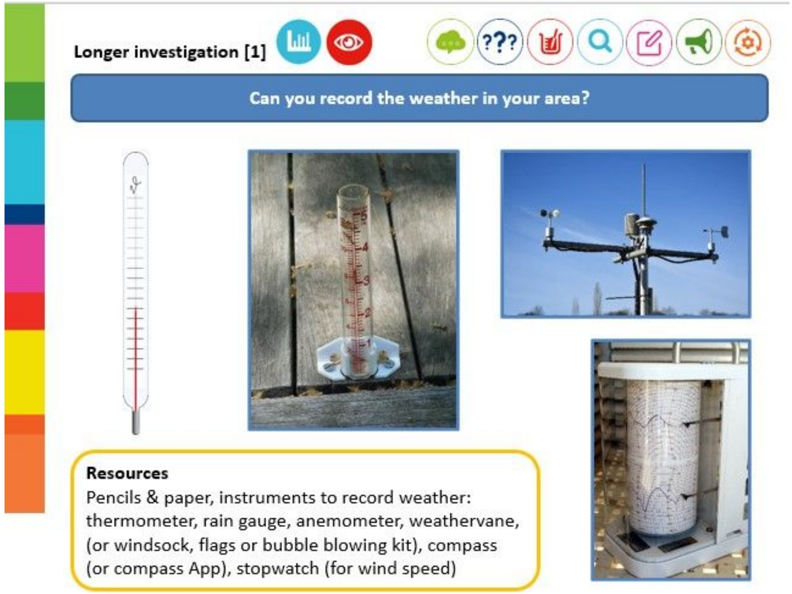

Figure S1. Introduction to a practical investigation.

Slide 3
Notes for Teachers – this slide is suitable for ages 4-11.

Background information for teachers
In 1854, Admiral Robert Fitzroy (Charles Darwin's captain on HMS Beagle) established what we now call the Met Office. For over 150 years, meteorologists (scientists who study the weather) have built up their understanding of the Earth's climate and weather patterns. They collect data including temperature, atmospheric pressure and wind speed and direction in weather stations to help them make short- and long-range forecasts. Accurate forecasting of the weather has often played a pivotal role in history. Most notable was when D-Day was delayed by 24 hours to make the most of a predicted break in the stormy weather on the 6th of June 1944.

Possible learning outcomes
- Appreciate the nature, processes and methods of science - how meteorologists predict the weather
- Understand the uses and implications of science today and in the future, - how weather forecasts help people such as farmers, fishermen, events planners everyday, and are informing scientists and world leaders about climate change.

Key vocabulary - some of the words in this list are more suitable for older children.* Teachers should decide which words in this list are suitable for the ages and abilities of children in their class.
- Weather
- Temperature
- *Air pressure
- Wind/wind speed
- Rain/rainfall
- Etc…

Questions to ask children.
- **How do you think that scientists predict the weather?** Meteorologists use weather measurements (data) collected at

Slide 13
Making a weather station & recording the weather - suitable for ages 4-11

Possible learning outcomes

- Knowledge – Observe (over time), and describe the weather associated with the seasons
- Skills – predict, set up equipment, observe & measure, record, communicate, evaluate the methods

Key vocabulary – teachers should select words appropriate to the ages and ability of the children in their class (the words towards the end of the list are more suitable for older children)
Temperature
Thermometer – instrument to measure how warm/cold it is
Rain
Rain gauge – an instrument to collect and measure rainfall
Wind
Wind speed
Wind direction
Anemometer – an instrument to measure wind speed
Air pressure
Barometer – an instrument to measure the pressure around us

Questions to ask children

- **What parts of the weather can we measure?**
- **How will you do this?**
- **How will you record your measurements/data?**

What to do

- Ask the children for their ideas. You might want to organise groups to monitor one aspect of the weather for a period of time.
- Provide thermometers, rain gauges (the children could make these), and if you have them, an anemometer and a weathervane. You could investigate the direction of the wind and wind speed using bubbles, a compass and a stopwatch (see Met Office website below).
- Discuss how children will record their data. Older children could create graphs showing variations over time in temperature, rainfall, wind speed and even air pressure. Teachers working with younger children could create class graphs.

Useful websites
There are lots of related activities suitable for ages 7-11 on the Met Office website, including:

- How to make a barometer - https://www.metoffice.gov.uk/weather/learn-about/met-office-for-schools/themes-for-7-11/forecasting-and-prediction
- How to measure wind speed and direction using bubbles - https://www.metlink.org/fieldwork-resource/bubble-chase/

Figure S2. Accompanying slide notes for each slide provide possible learning objectives that would align with many primary science curricula.

Article	Title	Curriculum Focus	Published	URL	Number of article downloads to Sep 23
1	How to calculate the age of a shark	Growing older	Oct-17	https://pstt.org.uk/download/2890/?tmstv=1677081279	1975
2	How to grow a new skin	Human body	Feb-18	https://pstt.org.uk/download/2887/?tmstv=1677081195	921
3	Planetary hide and seek	Hidden planets in space	Jun-18	https://pstt.org.uk/download/2883/?tmstv=1677080711	1016
4	How to clean water using a molecular sieve	Separating mixtures	Oct-18	https://pstt.org.uk/download/2877/?tmstv=1679326686	1204
5	Stripes and concealment	Adaptation (camouflage)	Feb-19	https://pstt.org.uk/download/2874/?tmstv=1677079872	1224
6	How plants know good microbes from bad ones	Chemical changes	Jun-19	https://pstt.org.uk/download/2870/?tmstv=1677079766	653
7	One of Saturn's moons may be a home for terrestrial life	Life in space	Sep-19	https://pstt.org.uk/download/2864/?tmstv=1677079556	844
8	The disastrous effects of historical ink	Separating materials (ink)	Oct-19	https://pstt.org.uk/download/6993/?tmstv=1695910331	694
9	Computers can measure the happiness of a city	Gases/Air pollution	Nov-19	https://pstt.org.uk/download/2857/?tmstv=1677079066	1048
10	Slug slime might be the answer for	Properties and uses of materials (adhesives)	Dec-19	https://pstt.org.uk/download/2851/?tmstv=1677078814	977

	medical adhesives				
11	Miracle healing could come from the axolotl	DNA code	Jan-20	https://pstt.org.uk/download/2848/?tmstv=1677078480	985
12	Whale song is changing	Sound (pitch/loudness)	Feb-20	https://pstt.org.uk/download/2845/?tmstv=1677078317	1514
13	Catching flu might depend on where you live	Staying healthy (viruses)	Mar-20	https://pstt.org.uk/download/2835/?tmstv=1677072234	69
14	Dragons could save us from 'bad' bacteria	Staying healthy (bacteria)	Mar-20	https://pstt.org.uk/download/2839/?tmstv=1677072340	856
15	What is happening to the bees	Insect pollination	Apr-20	https://pstt.org.uk/download/2831/?tmstv=1677072138	1635
16	Toilets in the future may charge your mobile phone	Separating materials	May-20	https://pstt.org.uk/download/2825/?tmstv=1677071995	1065
17	The science of hand-washing	Properties of materials	May-20	https://pstt.org.uk/download/2822/?tmstv=1677071909	1440
18	What small magnetic robots can do	Magnetic forces<br>Friction<br>Chemical change	May-20	https://pstt.org.uk/download/2819/?tmstv=1677071701	1109
19	Evolution of life in cities	Adaptation & evolution	Jun-20	https://pstt.org.uk/download/2813/?tmstv=1677071573	840
20	Blood tests could detect cancers	Human body parts<br>Disease	Jun-20	https://pstt.org.uk/download/2809/?tmstv=1677071439	797
21	Water can be harvested from the air in very dry climates	The water cycle	Jul-20	https://pstt.org.uk/download/2806/?tmstv=1677071024	1079

22	What happens underground when humans stay indoors	Vibrations	Sep-20	https://pstt.org.uk/download/2803/?tmstv=1676996665	982
23	Some mammals have unusual backbones	Skeletons and bones	Oct-20	https://pstt.org.uk/download/2800/?tmstv=1676996569	1178
24	Bacteria can get viral infections as well	Microbes	Nov-20	https://pstt.org.uk/download/2794/?tmstv=1676996479	835
25	Insight into Mars	Conditions on Mars	Dec-20	https://pstt.org.uk/download/2776/?tmstv=1676995998	1178
26	Ice giants at the edge of the Solar System	Giant planets and planetary motion	Dec-20	https://pstt.org.uk/download/2779/?tmstv=1676996104	918
27	The is a storm coming and it is not going away	Weather (storms)	Dec-20	https://pstt.org.uk/download/2787/?tmstv=1676996225	733
28	There is lightning at the edge of the Solar System	Weather (storms)	Dec-20	https://pstt.org.uk/download/2790/?tmstv=1676996360	928
29	Bees and caterpillars can change the evolution of plants	Adaptation and evolution	Jan-21	https://pstt.org.uk/download/2769/?tmstv=1676995900	1084
30	About biodiversity and trophic cascades	Food chains and webs	Feb-21	https://pstt.org.uk/download/2766/?tmstv=1676995790	1411
31	Termites can help rainforests survive	Food chains<br>Climate change	Mar-21	https://pstt.org.uk/download/2763/?tmstv=1676995175	1429

32	Tree restoration - it's now or never	Importance of trees<br>Climate change	Apr-21	https://pstt.org.uk/download/2757/?tmstv=1676995004	1819
33	Red squirrels adapt to the food they eat	Adaptation (diet/teeth)	May-21	https://pstt.org.uk/download/2753/?tmstv=1676994751	699
34	Which face mask you should wear	Properties and uses of materials (fabrics)	Jun-21	https://pstt.org.uk/download/2749/?tmstv=1676994610	825
35	Can plants hear and respond to sounds?	Adaptations (plants)	Jul-21	https://pstt.org.uk/download/2743/?tmstv=1676994391	564
36	Bamboo could be used to make cricket bats	Properties and uses of materials (bamboo)<br>Sustainability	Oct-21	https://pstt.org.uk/download/2725/?tmstv=1676994255	2688
37	Plastics in soil affect the survival of plants and worms	Germination and plant growth	Oct-21	https://pstt.org.uk/download/2720/?tmstv=1676994097	461
38	It's raining all over the world - extreme weather connections	Weather<br>Climate change	Nov-21	https://pstt.org.uk/download/2717/?tmstv=1676993941	461
39	We have telescopes out in space studying the universe	Light	Jan-22	https://pstt.org.uk/download/2713/?tmstv=1676993785	604
40	Why & how scientist measure mammals' whiskers	Adaptation (whiskers)	Feb-22	https://pstt.org.uk/download/2707/?tmstv=1676987945	271
41	The fossilised secrets of the rhino and the beetle	Fossils	Apr-22	https://pstt.org.uk/download/2704/?tmstv=1676987796	222

42	Floral scents may be changed by air pollutants	Adaptation (pollinating insects)	May-22	https://pstt.org.uk/download/2698/?tmstv=1676987197	148
43	Soil fungi could reduce global warming	Importance of soil and fungi	Oct-22	https://pstt.org.uk/download/5041/?tmstv=1679324643	145
44	Weird wiggly crawling wheels roam Mars	Understanding friction	Mar-23	https://pstt.org.uk/download/5705/?tmstv=1681296245	104
45	Geoengineering could slow the melting of Arctic Ice	Properties and uses of materials (reflectivity)	Jun-23	https://pstt.org.uk/download/7081/?tmstv=1698234193	108

Table S1. Summary table of all IBYDK articles written as of November 2023 detailing title, curriculum focus, URL and number of downloads.

REFERENCES

S1. Shallcross, D. E.; Ellis R. It's raining all over the world – Extreme weather connections. I bet you didn't know… https://pstt.org.uk/resources/curriculum-materials/cutting-edge-science-primary-schools (accessed Jan 23).

S2. Boers, N.; Goswami, B.; Rheinwalt, A.; Bookhagen, B.; Hoskins, B.; Kurths, J. Complex networks reveal global pattern of extreme-rainfall teleconnections. *Nature*. **2019**, 566, 373-377. https://doi.org/10.1038/s41586-018-0872-x (accessed Jan 2023)
