# Supplementary material for: Chemical Science Research, Elementary School Children and Their Teachers Are More Closely Related than You May Imagine: The “I Bet You Did Not Know” Project
Source: J Chem Educ. 2024 Jan 19;101(2):337–43. doi: 10.1021/acs.jchemed.3c00233 (PMC10867834; doi:10.1021/acs.jchemed.3c00233)
Supplement: Supplementary file 2 — ed3c00233_si_002.docx [file ed3c00233_si_002.docx]

Supporting Information for the paper

Chemical Science Research, Elementary School Children and their Teachers are more closely related than you may imagine; The ‘I bet you didn’t know’ project.

Alison J. Trew^1*^, Craig Early^1^, Rebecca Ellis^1^, Julia Nash^1^, Katharine Pemberton^1^, Paul Tyler^1^, Timothy G. Harrison^2#^ & Dudley E. Shallcross^2,3*^

1. Primary Science Teaching Trust. 12 Whiteladies Road, Bristol, BS8 1PD, U.K.
2. School of Chemistry, Cantock’s Close, University of Bristol, Bristol, BS8 1TS, UK.
3. Department of Chemistry, University of the Western Cape, Robert Sobukwe Road, Bellville, 7535, South Africa,

Corresponding authors Alison J. Trew (alison.trew@pstt.org.uk) and Dudley E Shallcross (d.e.shallcross@bris.ac.uk)

Overview

We present an extended description of the teacher guide that accompanies the exemplar article ‘It is raining all over the world’ and a table summarising all the articles written as of November 2023 and weblinks correct at the time of submission.

Extreme weather teacher guide example^S1,S2^

Figure S1 provides teaching input on new subject knowledge.

**
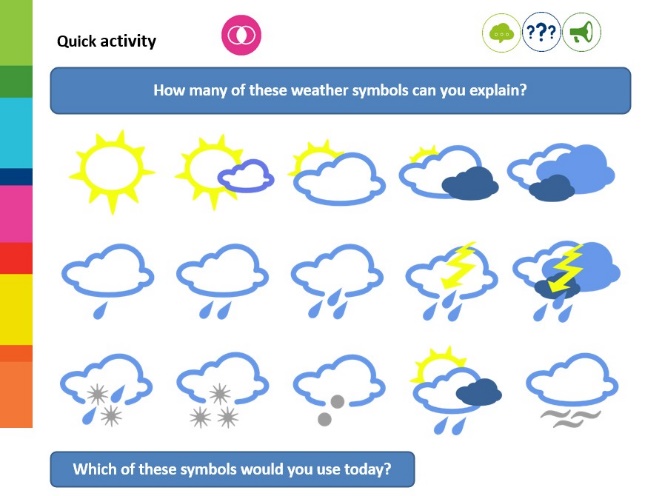

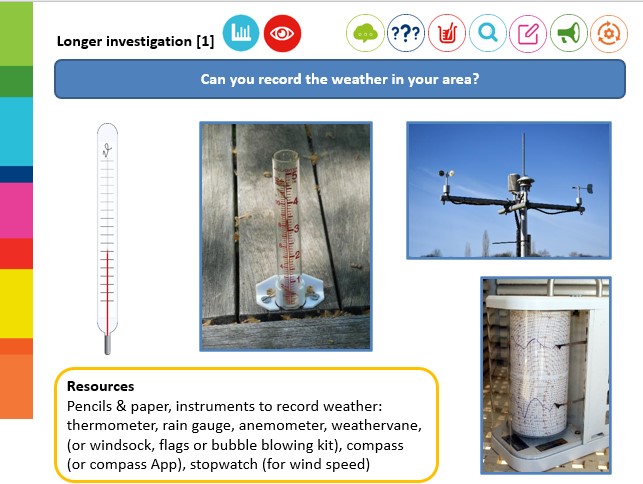
**

Figure S1. Introduction to a practical investigation.

**Slide 3**

**Notes for Teachers** – this slide is suitable for ages 4-11.

**Background information for teachers**

In 1854, Admiral Robert Fitzroy (Charles Darwin’s captain on HMS Beagle) established what we now call the Met Office. For over 150 years, meteorologists (scientists who study the weather) have built up their understanding of the Earth’s climate and weather patterns. They collect data including temperature, atmospheric pressure and wind speed and direction in weather stations to help them make short- and long-range forecasts. Accurate forecasting of the weather has often played a pivotal role in history. Most notable was when D-Day was delayed by 24 hours to make the most of a predicted break in the stormy weather on the 6th of June 1944.

**Possible learning outcomes**

- Appreciate the nature, processes and methods of science - how meteorologists predict the weather
- Understand the uses and implications of science today and in the future, - how weather forecasts help people such as farmers, fishermen, events planners everyday, and are informing scientists and world leaders about climate change.

**Key vocabulary -** some of the words in this list are more suitable for older children.* Teachers should decide which words in this list are suitable for the ages and abilities of children in their class.

- Weather
- Temperature
- *Air pressure
- Wind/wind speed
- Rain/rainfall
- Etc…

**Questions to ask children.**

- **How do you think that scientists predict the weather?** Meteorologists use weather measurements (data) collected at weather stations all across the country and computers to help them predict what will happen in the next few days.
- **What do you think a weather station measures?** Weather stations measure many different aspects of the weather including air temperature, the pressure caused by the weight of air particles above the Earth (atmospheric pressure) wind speed and direction, rainfall, how much water there is in the air (humidity), cloud height and visibility.
- **How do weather forecasts help us?** It helps people doing different jobs outside to plan what to do when and what equipment they need, e.g., farmers, fishermen, people planning sports events. You might like to share slide 20 to explain what once happened when a weather forecast was wrong.

**Slide 13**

**Making a weather station & recording the weather** - suitable for ages 4-11

**Possible learning outcomes**

- Knowledge – Observe (over time), and describe the weather associated with the seasons
- Skills – predict, set up equipment, observe & measure, record, communicate, evaluate the methods

**Key vocabulary** – teachers should select words appropriate to the ages and ability of the children in their class (the words towards the end of the list are more suitable for older children)

Temperature

Thermometer – instrument to measure how warm/cold it is

Rain

Rain gauge – an instrument to collect and measure rainfall

Wind

Wind speed

Wind direction

Anemometer – an instrument to measure wind speed

Air pressure

Barometer – an instrument to measure the pressure around us

**Questions to ask children**

- **What parts of the weather can we measure?**
- **How will you do this?**
- **How will you record your measurements/data?**

**What to do**

- Ask the children for their ideas. You might want to organise groups to monitor one aspect of the weather for a period of time.
- Provide thermometers, rain gauges (the children could make these), and if you have them, an anemometer and a weathervane. You could investigate the direction of the wind and wind speed using bubbles, a compass and a stopwatch (see Met Office website below).
- Discuss how children will record their data. Older children could create graphs showing variations over time in temperature, rainfall, wind speed and even air pressure. Teachers working with younger children could create class graphs.

**Useful websites**

There are lots of related activities suitable for ages 7-11 on the Met Office website, including:

- How to make a barometer - https://www.metoffice.gov.uk/weather/learn-about/met-office-for-schools/themes-for-7-11/forecasting-and-prediction
- How to measure wind speed and direction using bubbles - https://www.metlink.org/fieldwork-resource/bubble-chase/

Figure S2. Accompanying slide notes for each slide provide possible learning objectives that would align with many primary science curricula.

| **Article** | **Title** | **Curriculum Focus** | **Published** | **URL** | **Number of article downloads to Sep 23** |
| --- | --- | --- | --- | --- | --- |
| 1 | How to calculate the age of a shark | Growing older | Oct-17 | <https://pstt.org.uk/download/2890/?tmstv=1677081279> | 1975 |
| 2 | How to grow a new skin | Human body | Feb-18 | <https://pstt.org.uk/download/2887/?tmstv=1677081195> | 921 |
| 3 | Planetary hide and seek | Hidden planets in space | Jun-18 | <https://pstt.org.uk/download/2883/?tmstv=1677080711> | 1016 |
| 4 | How to clean water using a molecular sieve | Separating mixtures | Oct-18 | <https://pstt.org.uk/download/2877/?tmstv=1679326686> | 1204 |
| 5 | Stripes and concealment | Adaptation (camouflage) | Feb-19 | <https://pstt.org.uk/download/2874/?tmstv=1677079872> | 1224 |
| 6 | How plants know good microbes from bad ones | Chemical changes | Jun-19 | <https://pstt.org.uk/download/2870/?tmstv=1677079766> | 653 |
| 7 | One of Saturn's moons may be a home for terrestrial life | Life in space | Sep-19 | <https://pstt.org.uk/download/2864/?tmstv=1677079556> | 844 |
| 8 | The disastrous effects of historical ink | Separating materials (ink) | Oct-19 | <https://pstt.org.uk/download/6993/?tmstv=1695910331> | 694 |
| 9 | Computers can measure the happiness of a city | Gases/Air pollution | Nov-19 | <https://pstt.org.uk/download/2857/?tmstv=1677079066> | 1048 |
| 10 | Slug slime might be the answer for medical adhesives | Properties and uses of materials (adhesives) | Dec-19 | <https://pstt.org.uk/download/2851/?tmstv=1677078814> | 977 |
| 11 | Miracle healing could come from the axolotl | DNA code | Jan-20 | <https://pstt.org.uk/download/2848/?tmstv=1677078480> | 985 |
| 12 | Whale song is changing | Sound (pitch/loudness) | Feb-20 | <https://pstt.org.uk/download/2845/?tmstv=1677078317> | 1514 |
| 13 | Catching flu might depend on where you live | Staying healthy (viruses) | Mar-20 | <https://pstt.org.uk/download/2835/?tmstv=1677072234> | 69 |
| 14 | Dragons could save us from 'bad' bacteria | Staying healthy (bacteria) | Mar-20 | <https://pstt.org.uk/download/2839/?tmstv=1677072340> | 856 |
| 15 | What is happening to the bees | Insect pollination | Apr-20 | <https://pstt.org.uk/download/2831/?tmstv=1677072138> | 1635 |
| 16 | Toilets in the future may charge your mobile phone | Separating materials | May-20 | <https://pstt.org.uk/download/2825/?tmstv=1677071995> | 1065 |
| 17 | The science of hand-washing | Properties of materials | May-20 | <https://pstt.org.uk/download/2822/?tmstv=1677071909> | 1440 |
| 18 | What small magnetic robots can do | Magnetic forces  Friction  Chemical change | May-20 | <https://pstt.org.uk/download/2819/?tmstv=1677071701> | 1109 |
| 19 | Evolution of life in cities | Adaptation & evolution | Jun-20 | <https://pstt.org.uk/download/2813/?tmstv=1677071573> | 840 |
| 20 | Blood tests could detect cancers | Human body parts  Disease | Jun-20 | <https://pstt.org.uk/download/2809/?tmstv=1677071439> | 797 |
| 21 | Water can be harvested from the air in very dry climates | The water cycle | Jul-20 | <https://pstt.org.uk/download/2806/?tmstv=1677071024> | 1079 |
| 22 | What happens underground when humans stay indoors | Vibrations | Sep-20 | <https://pstt.org.uk/download/2803/?tmstv=1676996665> | 982 |
| 23 | Some mammals have unusual backbones | Skeletons and bones | Oct-20 | <https://pstt.org.uk/download/2800/?tmstv=1676996569> | 1178 |
| 24 | Bacteria can get viral infections as well | Microbes | Nov-20 | <https://pstt.org.uk/download/2794/?tmstv=1676996479> | 835 |
| 25 | Insight into Mars | Conditions on Mars | Dec-20 | <https://pstt.org.uk/download/2776/?tmstv=1676995998> | 1178 |
| 26 | Ice giants at the edge of the Solar System | Giant planets and planetary motion | Dec-20 | <https://pstt.org.uk/download/2779/?tmstv=1676996104> | 918 |
| 27 | The is a storm coming and it is not going away | Weather (storms) | Dec-20 | <https://pstt.org.uk/download/2787/?tmstv=1676996225> | 733 |
| 28 | There is lightning at the edge of the Solar System | Weather (storms) | Dec-20 | <https://pstt.org.uk/download/2790/?tmstv=1676996360> | 928 |
| 29 | Bees and caterpillars can change the evolution of plants | Adaptation and evolution | Jan-21 | <https://pstt.org.uk/download/2769/?tmstv=1676995900> | 1084 |
| 30 | About biodiversity and trophic cascades | Food chains and webs | Feb-21 | <https://pstt.org.uk/download/2766/?tmstv=1676995790> | 1411 |
| 31 | Termites can help rainforests survive | Food chains  Climate change | Mar-21 | <https://pstt.org.uk/download/2763/?tmstv=1676995175> | 1429 |
| 32 | Tree restoration - it's now or never | Importance of trees  Climate change | Apr-21 | <https://pstt.org.uk/download/2757/?tmstv=1676995004> | 1819 |
| 33 | Red squirrels adapt to the food they eat | Adaptation (diet/teeth) | May-21 | <https://pstt.org.uk/download/2753/?tmstv=1676994751> | 699 |
| 34 | Which face mask you should wear | Properties and uses of materials (fabrics) | Jun-21 | <https://pstt.org.uk/download/2749/?tmstv=1676994610> | 825 |
| 35 | Can plants hear and respond to sounds? | Adaptations (plants) | Jul-21 | <https://pstt.org.uk/download/2743/?tmstv=1676994391> | 564 |
| 36 | Bamboo could be used to make cricket bats | Properties and uses of materials (bamboo)  Sustainability | Oct-21 | <https://pstt.org.uk/download/2725/?tmstv=1676994255> | 2688 |
| 37 | Plastics in soil affect the survival of plants and worms | Germination and plant growth | Oct-21 | <https://pstt.org.uk/download/2720/?tmstv=1676994097> | 461 |
| 38 | It's raining all over the world - extreme weather connections | Weather  Climate change | Nov-21 | <https://pstt.org.uk/download/2717/?tmstv=1676993941> | 461 |
| 39 | We have telescopes out in space studying the universe | Light | Jan-22 | <https://pstt.org.uk/download/2713/?tmstv=1676993785> | 604 |
| 40 | Why & how scientist measure mammals' whiskers | Adaptation (whiskers) | Feb-22 | <https://pstt.org.uk/download/2707/?tmstv=1676987945> | 271 |
| 41 | The fossilised secrets of the rhino and the beetle | Fossils | Apr-22 | <https://pstt.org.uk/download/2704/?tmstv=1676987796> | 222 |
| 42 | Floral scents may be changed by air pollutants | Adaptation (pollinating insects) | May-22 | <https://pstt.org.uk/download/2698/?tmstv=1676987197> | 148 |
| 43 | Soil fungi could reduce global warming | Importance of soil and fungi | Oct-22 | <https://pstt.org.uk/download/5041/?tmstv=1679324643> | 145 |
| 44 | Weird wiggly crawling wheels roam Mars | Understanding friction | Mar-23 | <https://pstt.org.uk/download/5705/?tmstv=1681296245> | 104 |
| 45 | Geoengineering could slow the melting of Arctic Ice | Properties and uses of materials (reflectivity) | Jun-23 | <https://pstt.org.uk/download/7081/?tmstv=1698234193> | 108 |

Table S1. Summary table of all IBYDK articles written as of November 2023 detailing title, curriculum focus, URL and number of downloads.

**REFERENCES**

S1. Shallcross, D. E.; Ellis R. It’s raining all over the world – Extreme weather connections. I bet you didn’t know… <https://pstt.org.uk/resources/curriculum-materials/cutting-edge-science-primary-schools> (accessed Jan 23).

S2. Boers, N.; Goswami, B.; Rheinwalt, A.; Bookhagen, B.; Hoskins, B.; Kurths, J. Complex networks reveal global pattern of extreme-rainfall teleconnections. *Nature*. **2019**, 566, 373-377. <https://doi.org/10.1038/s41586-018-0872-x> (accessed Jan 2023)
